# A new mechanism of trastuzumab resistance in gastric cancer: MACC1 promotes the Warburg effect via activation of the PI3K/AKT signaling pathway

**DOI:** 10.1186/s13045-016-0302-1

**Published:** 2016-08-31

**Authors:** Jing Liu, Changqie Pan, Lihong Guo, Mengwan Wu, Jing Guo, Sheng Peng, Qianying Wu, Qiang Zuo

**Affiliations:** 1 Department of Oncology, Nanfang Hospital, Southern Medical University, Guangzhou, Guangdong Province China; 2 Department of ICU, Zhujiang Hospital, Southern Medical University, Guangzhou, Guangdong Province China

**Keywords:** Metastasis-associated in colon cancer-1, Trastuzumab, Resistance, Warburg effect, PI3K/AKT signaling pathway, Gastric cancer

## Abstract

**Background:**

Trastuzumab, a humanized antibody targeting HER2, exhibits remarkable therapeutic efficacy against HER2-positive gastric cancer. However, recurrent therapeutic resistance presents revolutionary claims. Warburg effect and AKT signaling pathway was involved in the resistance to trastuzumab. Our previous studies have demonstrated that overexpression of metastasis associated with the colon cancer 1 (MACC1) predicted poor prognosis of GC and promoted tumor cells proliferation and invasion. In this study, we found that MACC1 was significantly upregulated in trastuzumab-resistant cell lines. Besides, downregulation of MACC1 reversed this resistance.

**Methods:**

The effect of trastuzumab and glycolysis inhibitor combination on cell viability, apoptosis, and cell metabolism was investigated in vitro using established trastuzumab-resistant GC cell lines. We assessed the impact of trastuzumab combined with oxamate on tumor growth and metabolism in an established xenograft model of HER2-positive GC cell lines.

**Results:**

Here, we found that MACC1 was significantly upregulated in trastuzumab-resistant cell lines. Besides, downregulation of MACC1 in trastuzumab-resistant cells reversed this resistance. Overexpression of MACC1-induced trastuzumab resistance, enhanced the Warburg effect, and activated the PI3K/AKT signaling pathway, while downregulation of MACC1 presented the opposite effects. Moreover, when the PI3K/AKT signaling pathway was inhibited, the effects of MACC1 on resistance and glycolysis were diminished. Our findings indicated that MACC1 promoted the Warburg effect mainly through the PI3K/AKT signaling pathway, which further enhanced GC cells trastuzumab resistance.

**Conclusions:**

Our results indicate that co-targeting of HER2 and the Warburg effect reversed trastuzumab resistance in vitro and in vivo, suggesting that the combination might overcome trastuzumab resistance in MACC1-overexpressed, HER2-positive GC patients.

**Electronic supplementary material:**

The online version of this article (doi:10.1186/s13045-016-0302-1) contains supplementary material, which is available to authorized users.

## Background

Gastric cancer is the fifth common cancer and the third causes of tumor deaths worldwide [1]. Human epidermal growth factor receptor 2 (HER2) is a member of the receptor family associated with tumor cell proliferation, adhesion, migration, and differentiation. Trastuzumab, a humanized monoclonal antibody that targets HER2, inhibits the HER2-mediated signaling pathway and induces antibody-dependent cellular cytotoxicity [2]. For HER2-positive advanced gastric cancer (GC) patients, combining chemotherapy with trastuzumab is significantly superior to chemotherapy alone with regard to efficacy and safety [3, 4]. Although the response rates to this combination are far higher than those of chemotherapy alone, the effects are usually transitory, suggesting a high incidence of resistance [4–6]. Thus, more effective predictors of trastuzumab response in HER2-positive cancer, except for HER2, are required for personalized clinical treatment.

Cancer cells prefer anaerobic breakdown of glucose for energy rather than mitochondrial oxidative phosphorylation, this phenomenon termed “Warburg effect” [7]. The Warburg effect, which is the most common metabolic phenotype in cancer cells, has been closely correlated with drug resistance in cancer cells [8–10]. Since now, many glycolysis inhibitors have been evaluated to overcome the anticancer therapy resistance, such as MTCI inhibitors [11], PDK inhibitors [12], lactate dehydrogenase A (LDHA) inhibitors, and HK inhibitors [13]. Several glycolysis inhibitors are currently under pre-clinical and clinical researches. 2-Deoxy-d-glucose (2-DG), is a glucose analog that inhibits glycolysis via its actions on hexokinase, presented a tolerable adverse effects in combination usage with docetaxel [14]. Oxamate, a specific inhibitor of the lactate dehydrogenase, is the most promising target to develop glycolysis inhibitors with selective activity on cancer cells because of its unnecessary for normal tissue surviva [15]. Therefore, appropriate combining glycolysis inhibitor with anticancer reagents might be a key for overcoming the drug resistance.

One of the major mechanisms underlying trastuzumab resistance in breast cancer is the dysregulation of HER2 downstream signaling substrate, including the phosphatidylinositol 3-kinase/protein kinase B (PI3K/AKT) pathway [16, 17]. We recently found that activation of the PI3K/AKT signaling pathway can leading to resistance of HER2-positive GC cells to trastuzumab [18]. As AKT activation stimulates aerobic glycolysis in both solid tumors and cancers of hematopoietic directly [19], it is reasonable to hypothesize that activation of AKT might induce enhancement of Warburg effect and resulted in trastuzumab resistance in GC cells.

Metastasis associated in the colon cancer 1 (MACC1) gene which was identified by Stein et al. is demonstrated to be upregulated in several types of cancer and served as a biomarker for cancer invasion and metastasis [20]. Previously, we found that MACC1 contributed to poor prognosis of GC by promoting tumor cells proliferation and invasion as well as epithelial-to-mesenchymal transition [21]. Moreover, we discovered that MACC1 was upregulated by metabolic stress in GC via adenosine monophosphate-activated protein kinase signaling, which increased the resistance to metabolic stress by promoting the Warburg effect and consequently facilitated tumor progression [22]. MACC1 is a regulator of MET/AKT signaling pathway which has also been approved by our previous work [23].

Here, we first found that MACC1 was significantly upregulated in trastuzumab-resistant NCI-N87/TR and MKN45/TR cell lines. MACC1 promoted the Warburg effect mainly via the PI3K/AKT signaling pathway, which further enhanced the resistance to trastuzumab. To clarify this mechanism, we herein investigated the relationship between MACC1 expression, the Warburg effect, and trastuzumab resistance in HER2-positive GC cells. Taken together, these findings provide evidence for unraveling the mechanism of trastuzumab resistance and improving the efficacy of treatment.

## Methods

### Cell lines and culture conditions

Human gastric cancer cells including SGC7901, MKN45, and NCI-N87 were obtained from the American Type Culture Collection (ATCC). BGC-823 and MKN-28 were obtained from Foleibao Biotechnology Development (Shanghai, China). Cells were cultured in complete medium (Roswell Park Memorial Institute 1640 medium (Invitrogen, Life Technologies, Carlsbad, CA) with 10 % fetal bovine serum (Thermo Scientific HyClone, South Logan, UT)) and incubated under 5 % CO2 at 37 °C. Cells were collected in logarithmic growth phase for all experiments as described in the following sections.

### Antibodies and chemicals

MACC1-expressing and HER2-positive cells were selected using Western blotting analyses. Antibodies against the following proteins were used in this study: MACC1 (Abnova, Taipei, China), GLUT1 (Epitomics, Burlingame, CA, USA), hexokinase 2 (HK2), GAPDH and phosphorylation-AKT (Ser473) (Cell Signaling Technology, Danvers, MA, USA) and HER2, LDHA, and AKT (Abcam, USA). IHC staining was done with the Dako Envision System (Dako, Glostrup, Denmark). MitoTracker Red CMXRos (Invitrogen, Carlsbad, CA, USA) or 4,6-diamidino-2-phenylindole was stained when needed to label themitochondria or nucleus. Trastuzumab was supported by Roche company. AKT inhibitor MK2206 and PI3K inhibitor LY294002 were obtained from Calbiochem (Selleck Chemicals, USA), and the Warburg effect inhibitor sodium oxamate was obtained from Sigma-Aldrich (Shanghai Trading Co., Ltd., China). 2-Deoxy-d-glucose (2DG) was obtained from Biotechnology (Santa Cruz Biotechnology, Inc., California).

### Myr-AKT plasmids

The cDNA encoding myristoylated-human AKT lacking the PH domain (Myr-AKT) was cloned into the pCAGGS-IRESEGFPpA vector to produce the active AKT expression plasmid.

### Induction of trastuzumab-resistant NCI-N87/TR and MKN45/TR cells

The establishment of NCI-N87/TR has been previously described in our last study [18]. Aliquots of MKN45 cells in the exponential growth phase were seeded into 25 cm^2^ culture bottles. Trastuzumab was added for 48 h during the mitotic phase, and then, the cells were transferred into drug-free culture medium until the next mitotic phase, after which trastuzumab was added for the next 48 h at twice the previous concentration. We continued this process while observing cell death every day, changing to fresh complete culture medium, and performing the MTT assay regularly. This process was continued until the concentration of trastuzumab in the medium reached 2560 μg/ml after 150 days. Thus, MKN45 cells were obtained, which were grown stably in trastuzumab (2560 μg/ml)-containing medium, and these trastuzumab-resistant cells were named MKN45/TR cells.

### Establishment of stably transfected cell lines

For MACC1 overexpression, the ectopic MACC1 coding sequence was amplified by polymerase chain reaction (PCR) (primer sequences in Additional file 1: Figure S1b) and cloned into the pLVX-MCMV-ZsGreen-PGK-Puro plasmid. For MACC1 silencing, sequences of short hairpin RNA targeting MACC1 (shMACC1) and scramble were cloned into the pLVX plasmid sequences (Additional file 1: Figure S1b). Cell lines were transfected with these constructed plasmids combined with the blank vector. Stably transfected cell lines were selected with 0.5 mg/mL (a minimum lethal dose) puromycin at 48 h after infection. By this selection criterion, MACC1 expression was markedly increased in the MACC1 overexpression group and strongly inhibited in the MACC1 silencing group in the transfected GC cells.

### Transient transfection

Cells were transfected with various plasmids using Lipofectamine (Invitrogen) according to the manufacturer’s instructions, seeded onto 6-well plates (3 × 10^5^ cells per well) and incubated in 10 % FBS-containing medium for 24 h prior to drug treatment.

### Cell-based assay for glucose uptake and lactate assay

The levels of glucose uptake were measured using an Amplex® Red Glucose/Glucose Oxidase Assay Kit (Invitrogen). Cells were seeded in 96-well plates at a density of 5 × 10^3^ cells/well. After 24 h, glucose uptake assays were performed according to the manufacturer’s protocol. Relative fluorescence units were determined at 485–535 nm using a VARIOSKAN FLASH Multimode Reader (Thermo). The levels of lactate production were examined using a Lactate Colorimetric/Fluorometric Assay Kit (Biovision, Milpitas, CA, USA). Cells were plated in 96-well plates at a density of 5 × 10^3^ cells/well. After incubation for 24 h, the culture medium was replaced with FBS-free DMEM. After an additional 8 h, lactate assays were performed with culture media collected from each sample according to the manufacturer’s protocol, and the optical density was measured at 570 nm using Multiskan EX (Thermo).

### Western blotting analysis

Harvested cells were lysed with lysis buffer containing 50 mM Tris/HCl, pH 8.0, 150 mM NaCl, 1 % NP-40, 0.5 % sodium deoxycholate, 0.1 % SDS, 50 mM NaF, 1 mM Na_3_VO_4_, and protease inhibitor (Roche, Indianapolis, IN, USA). Protein concentrations in the cell lysates were quantified using the BCA protein assay kit (Thermo Scientific). Proteins were separated on SDS-PAGE gels and transferred onto nitrocellulose membrane (Whatman, Maidstone, UK). After being blocked with 5 % skim milk in TBS containing 0.05 % Tween-20, the membranes were incubated in 5 % skim milk containing the appropriate primary antibodies overnight, followed by incubation with horseradish peroxidase-conjugated secondary antibodies for 2 h. The protein bands were visualized using a commercial ECL kit (Beyotime, China).

### Cell viability assay

Cells were seeded onto 96-well plates at a density of 3 × 10^3^ cells/well. Twenty-four hours later, the cells were treated with drugs at the indicated concentrations and incubated for specific time periods. Cell viability was determined 3 days after treatment using the cell proliferation kit II (Roche Molecular Biochemicals) with the 3′-(4,5-dimethylthiazol-2-yl)-2,5-diphenyltetrazolium bromide (MTT) assay (Sigma, St. Louis, MO, USA) according to the manufacturer’s protocol. Experiments were performed in triplicate.

### Flow cytometric analysis of apoptosis

Cells were treated with trastuzumab for 48 h, collected, washed with phosphate-buffered saline (PBS) containing 0.1 % bovine serum albumin, and resuspended in 500 μl binding buffer. Next, the cells were incubated with 5 μl of Annexin V-FITC and 10 μl of PI solution for 15 min at room temperature in the dark. Subsequently, the samples were evaluated for apoptosis using a flow cytometer (FACS Calibur; BD Biosciences, Franklin Lakes, NJ, USA), Annexin V-FITC Apoptosis Detection Kit was bought from Biovision USA.

### Nude mice cancer xenograft model

All experimental procedures involving animals were performed according to the Guide for the Care and Use of Laboratory Animals (NIH publication no. 80-23, revised in 1996) and were performed in compliance with the institutional ethical guidelines for animal experimentation. GC cells were pre-treated with different plasmids or NC. The cells were suspended in 100 μl of PBS at a concentration of 5 × 10^7^ cells/ml and injected into either flank of the same BALB/C female athymic nude mouse at 5–6 weeks of age (six mice for each group, *n* = 6). The tumor size was monitored by measuring the length (*L*) and width (*W*) with calipers, and the volumes were calculated using the formula: (*L* × *W*^2^) × 0.5.

### Histology and immunohistochemistry

Mice were sacrificed with CO_2_, and half-dissected tumors were snap-frozen in liquid nitrogen to prepare protein lysates or were fixed in 10 % neutral-buffered formalin overnight at room temperature, transferred to 70 % ethanol, embedded in paraffin, and sectioned at 5 μm for IHC staining. Hematoxylin and eosin staining was performed in the Department of Pathology at Nanfang Hospital. IHC staining for MACC1 was performed using previously described methods [21].

After the animals were sacrificed, the formed tumors were harvested, fixed with formalin, and embedded in paraffin wax. Tissue was cut into 4-μm sections on clean, charged microscope slides, and then heated in a tissue-drying oven for 45 min at 60 °C. After deparaffinization, antigen retrieval was performed. The slides were incubated in 0.01 M sodium citrate buffer, pH 6.0 at 100 °C for 20 min, removed from the heat, cooled in buffer at room temperature for 20 min, and rinsed in 1× TBS with Tween at room temperature for 1 min. After being blocked in 5 % BSA, the section was incubated with diluted primary antibody at room temperature for 45 min, washed with 1× TBS with Tween three times, incubated with specific biotinylated secondary antibody at room temperature for 30 min, and finally color developed by the addition of substrates.

### MicroPET/CT

The xenograft-bearing mice were fasted overnight and anesthetized with inhaled isoflurane. ^18^F-FDG of about 200 μCi per mouse was injected into the tail vein. After 60 min of nonspecific clearance, the mouse was scanned in microPET/CT Inveon scanner (Siemens, Knoxville, TN, USA) and images were then reconstructed using a two-dimensional ordered subsets expectation maximization algorithm. PET and CT image fusion and image analysis were performed using software ASIPro 5.2.4.0 (Siemens).

### TUNEL

Terminal deoxynucleotidyl transferase dUTP nick end labeling (TUNEL) assays were performed on sections using an DeadEndTM Colorimetric TUNEL assay kit (Promega, Madison, USA) principally according to the supplier’s instruction.

### Chou-Talalay method

Using the CalcuSyn Version 2.11 (Copyright Biosoft, USA) software, the combination index (CI) was calculated for cells receiving combination therapy according to the Chou and Talalay mathematical model for drug interactions. The resulting CI theorem of Chou-Talalay offers quantitative definition for an additive effect (CI = 1), synergism (CI < 1), and antagonism (CI > 1) in drug combinations [24].

### Statistical analysis

All data were represented as the mean of at least triplicate samples ± standard deviation. Statistical analysis included one-way ANOVA or Student’s *t* test using SPSS 20.0. *P* values less than 0.05 were considered statistically significant.

## Results

### MACC1 contributed to the resistance of HER2-positive GC cells in response to trastuzumab

**Fig. 1:**
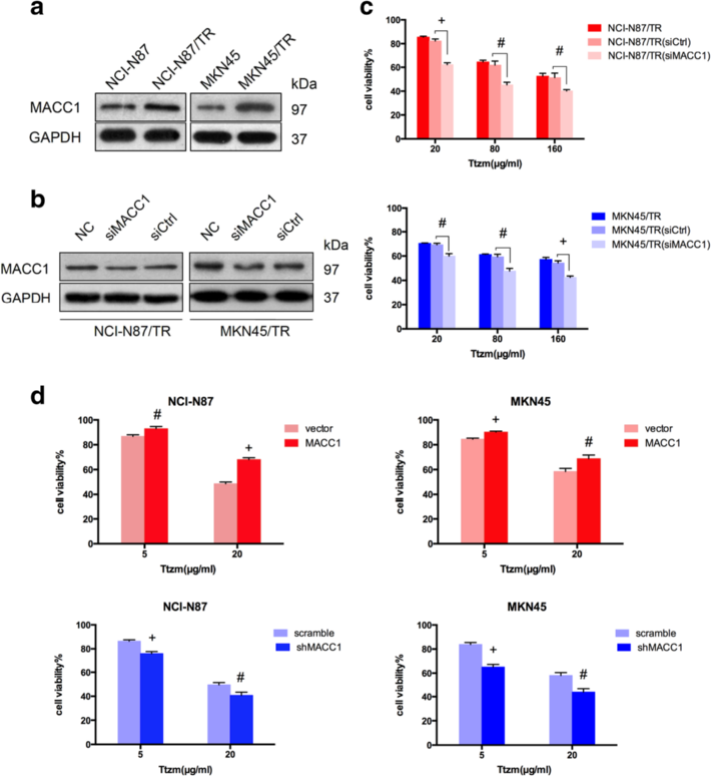
Effect of MACC1 on the resistance to trastuzumab in HER2-positive GC cell lines. **a** Western blot analysis of MACC1 expression in NCI-N87 and MKN45 parental cells and trastuzumab-resistant cells NCI-N87/TR and MKN45/TR. GAPDH was used as a loading control. **b** Western blot analysis of protein extracts from NCI-N87/TR and MKN45/TR cells 48 h after transient transfected by siMACC1 or siCtrl. NC, NCI-N87/TR, or MKN45/TR cells. GAPDH was used as a loading control. **c** Percentage of cell viability. MTT assays were performed 72 h after trastuzumab (Ttzm) treatment of the indicated cells at the different concentrations. Data represent mean ± SD of triplicate experiments relative to untreated cells. *P < 0.05, ^#^ *P* < 0.01, ^+^ *P* < 0.001. **d** Percentage of cell viability. MTT assays were performed 72 h after trastuzumab treatment of the indicated cells at the different concentrations. Data represent mean ± SD of triplicate experiments,*P < 0.05, ^#^ *P* < 0.01, ^+^ *P* < 0.001

In a previous study, we employed human gastric carcinoma cell line NCI-N87 with high HER2 expressions to generate trastuzumab-resistant NCI-N87/TR cell lines via stepwise exposure to increasing doses of trastuzumab [18]. Unexpectedly, compared with parental cells, the expression of MACC1 was significantly increased in trastuzumab-resistant cells (Fig. 1a).

To evaluated the effects of MACC1 on resistance to trastuzumab in GC cells, first, we tested the expression of HER2 and MACC1 protein levels (Additional file 1: Figure S1a) and the sensitivity to trastuzumab of MKN28, BGC823, SGC7901, MKN45, and NCI-N87 cell lines. Here, MKN45 cell line was chosen as the relatively sensitive cells to trastuzumab (Additional file 2: Table S1). We also established MKN45 trastuzumab-resistant cell line by stepwise exposure to increasing concentrations of trastuzumab (Additional file 2: Table S2) and found that MACC1 was also upregulated in MKN45/TR cells (Fig. 1a).

Next, to determine whether MACC1 was a regulatory factor in resistance to trastuzumab in HER2-positive GC cells, MACC1 was downregulated by small interfering RNA (siRNA) in NCI-N87/TR and MKN45/TR cell lines (Fig. 1b). Cell viability of the MACC1-downregulated cell lines was much more inhibited by trastuzumab than the resistant cells. Thus, targeting MACC1 reversed the trastuzumab resistance observed in HER2-positive GC cells (Fig. 1c).

To further identify the role of MACC1 in resistance to trastuzumab in HER2-positive GC cells, colonies of ectopic-MACC1 and shMACC1 and their respective controls were used to transfect NCI-N87 and MKN45 cells. Next, MACC1 overexpressing and downregulation NCI-N87 and MKN45 cells were treated with trastuzumab. Overexpression of MACC1 significantly increased the cell viability. Conversely, downregulation of MACC1 significantly inhibited the sensitivity of cells to trastuzumab (Fig. 1d). Collectively, these data indicated that MACC1 contributed to the resistance of HER2-positive GC cells to trastuzumab.

### MACC1 enhanced the Warburg effect in GC cells

**Fig. 2:**
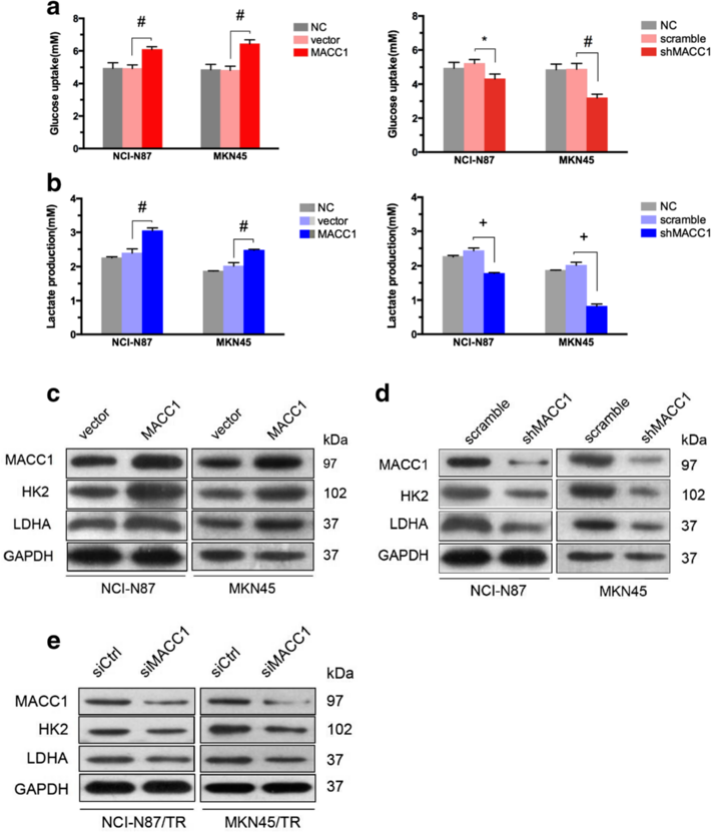
Enhancement effect of MACC1 on the Warburg effect in HER2-positive GC cell lines. **a, b** Glucose uptake (**a**) and lactate production (**b**) of the cells was measured 24 h after NCI-N87 and MKN45 MACC1 overexpression or downregulation cells and their control (vector and scramble). NC, NCI-N87, or MKN45 parental cells. Cells seeded in 96-well plates at 5 × 10^3^ cells/well, respectively. Data represent mean ± SD of triplicate experiments,*P < 0.05, ^#^ *P* < 0.01, ^+^ *P* < 0.001. **c** Western blot analysis of protein extracts from NCI-N87 and MKN45 cells 48 h after transient transfected by MACC1 or vector. GAPDH was used as a loading control. **d** Western blot analysis of protein extracts from NCI-N87 and MKN45 cells 48 h after transient transfected by siMACC1 or siCtrl. GAPDH was used as a loading control. **e** Western blot analysis of protein extracts from NCI-N87/TR and MKN45/TR cells 48 h after transient transfected by siMACC1 or siCtrl. GAPDH was used as a loading control

As our previously reported, MACC1 upregulation increased the resistance to metabolic stress by promoting the Warburg effect [16]. Since Warburg effect was closely correlated with trastuzumab resistance [25], we hypothesized that MACC1 may regulate resistance via Warburg effect. The levels of glucose uptake and lactate production were measured between the MACC1 upregulated and downregulated cells. The glucose uptake (Fig. 2a) and lactate production (Fig. 2b), which are hallmarks of glycolysis, obviously increased in MACC1-upregulated cells, while, decreased notably in MACC1-downregulated cells. In addition, the expression of HK2 and LDHA, which are rate-limited enzymes in the Warburg effect [26], decreased in MACC1-downregulated cells, whereas, increased in MACC1-upregulated cells (Fig. 2c, d). When MACC1 was silenced in NCI-N87/TR and MKN45/TR cells, the expression of HK2 and LDHA proteins were also downregulated (Fig. 2e). On the basis of our published paper [22] and these results, MACC1 enhances Warburg effect in GC cells.

### Synergistic cell growth and glycometabolism inhibition via the combination of trastuzumab and glycolysis inhibitor

**Fig. 3:**
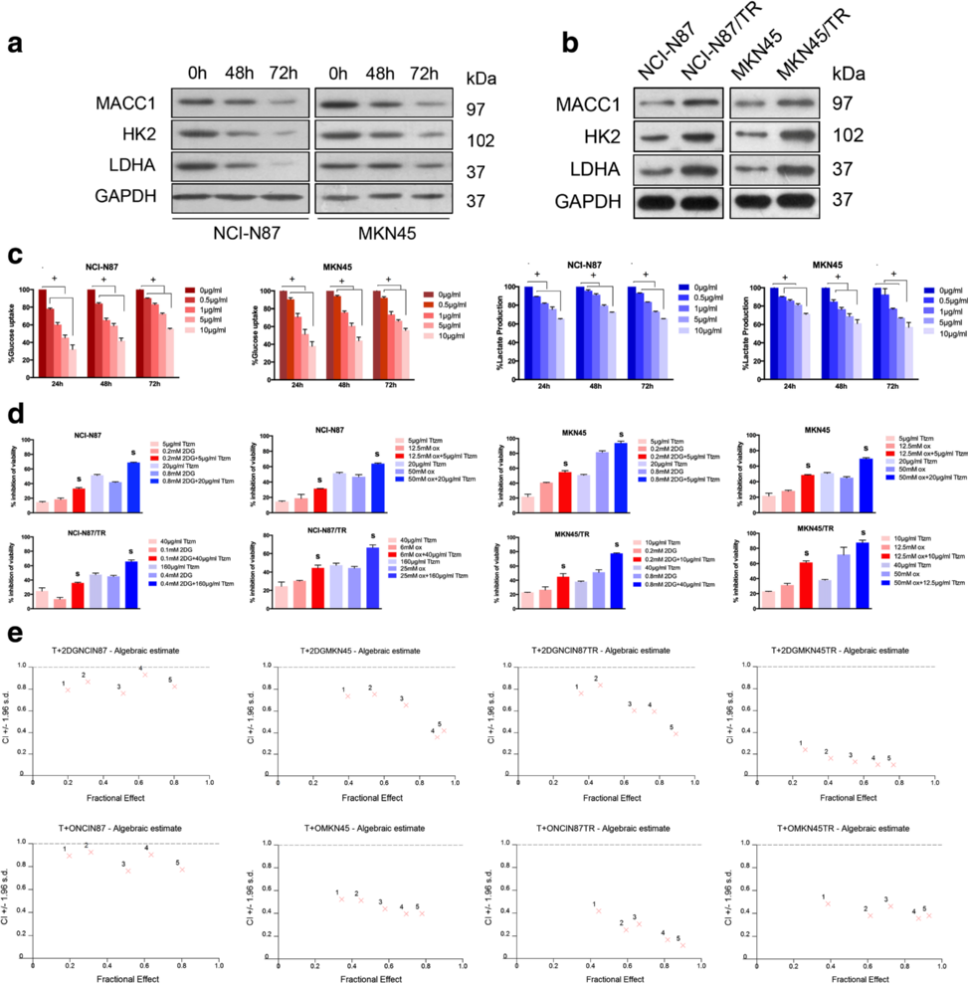
The combination of trastuzumab and glycolysis inhibitors results in a synergistic effect. **a** Western blot analysis of protein extracts from NCI-N87 and MKN45 cells 72 h after treated by Ttzm (40 μg/ml). GAPDH was used as a loading control. **b** Western blot analysis of MACC1, HK2, and LDHA expression in NCI-N87 and MKN45 parental cells and trastuzumab-resistant cells NCI-N87/TR and MKN45/TR. GAPDH was used as a loading control. **c** Percentage of glucose uptake (*red*) and lactate production (*blue*) of NCI-N87and MKN45 cells, 24, 48, and 72 h after Ttzm treatment at different concentrations. Data represent mean ± SD of triplicate experiments. Results are statistically significant for all the drugs vs. control by one-way ANOVA.*P < 0.05, ^#^ *P* < 0.01, ^+^ *P* < 0.001. **d** NCI-N87 and MKN45 parental (*upper*) and trastuzumab-resistant cells (*below*) were seeded in 96-well plates at 3 × 10^3^ cells/well. After 24 h, cells were treated with the indicated concentration of Ttzm, 2-deoxyglucose (2-DG), oxamate (OX), or Ttzm plus 2-DG/OX, incubated for 72 h and cell viability was determined. (The concentration of Ttam, 2-DG, and OX was represented in the Additional file 2: Table S3). Representative data are presented as the percentage of viability inhibition measured in cells not treated with Ttzm nor 2-DG/OX. Data represent mean ± SD of triplicate experiments,*P < 0.05, ^#^ *P* < 0.01, ^+^ *P* < 0.001. *S*, synergy (CI < 1.0). **e** Combination index (*CI*) for experimental values of cell viability inhibition by Ttzm plus 2-DG/OX in NCI-N87 and MKN45 parental cells and trastuzumab-resistant cells, as measured by Chou and Talalay method

Given that trastuzumab has been known to inhibited cell glycolysis in breast cancer cells [13], we test if it has the same effect in HER2-positive GC cells. NCI-N87 and MKN45 cells were treated with trastuzumab, HK2 and LDHA expression were downregulated after treatment with trastuzumab (Fig. 3a). Meanwhile, the glucose uptake and lactate production were also declined (Fig. 3c), indicating that trastuzumab effectively inhibits Warburg effect in GC cells.

Moreover, we found that when the GC cells became resistant to trastuzumab, the expression of HK2 and LDHA were highlighted (Fig. 3b). These results showed that the Warburg effect play an important role in trastuzumab resistance in GC cells, and it is logical to hypothesize that the combination of trastuzumab with glycolysis inhibitors may have more powerful antitumor effect than either agent alone [13].

To testify this hypothesis, we use the inhibitor of glycometabolism, 2-DG, or oxamate, combined with trastuzumab to treat both parental and trastuzumab-resistant GC cells. The combination of trastuzumab and 2-DG/oxamate showed a strongly increased inhibitory efficacy in both cell viabilities and glycometabolism of MKN45 and NCI-N87 parental cells and MKN45/TR and NCI-N87/TR cells compared to use the agents individually (Fig. 3d, Additional file 1: Figure S2). To evaluate whether the combination effect is synergistic or not, the date generated from NCI-N87, MKN45, NCI-N87/TR, and MKN45/TR cells treated with multiple concentrations of trastuzumab and 2-DG/oxamate were analyzed by the CalcuSyn software [24]. The combined index (CI) < 1.0 obtained in almost all combined treatment groups. Moreover, the synergistic effect in trastuzumab-resistant cells was more robust than in parental cells (Fig. 3e, Additional file 1: Figure S3, Additional file 2: Tables S3 and S4). With these data, we confirmed that the combination of trastuzumab with glycolysis inhibitors better suppressed cell viability and glycometabolism in HER2-positive GC cells, and this specific combination could overcome trastuzumab resistance.

### MACC1 promoted the Warburg effect via the PI3K/AKT signaling pathway and induced trastuzumab resistance in vitro

**Fig. 4:**
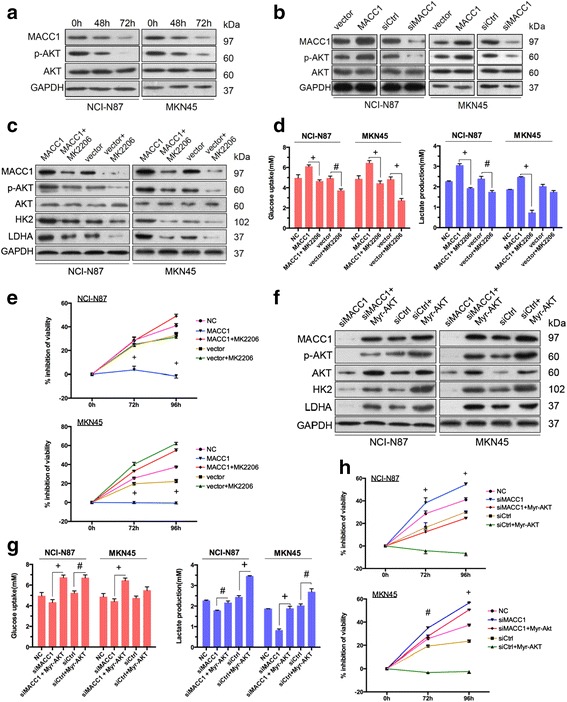
MACC1 promoted the Warburg effect and induced resistance to trastuzumab via PI3K/AKT pathway. **a** Western blot analysis of protein extracts from NCI-N87 and MKN45 cells 48 and 72 h after treated by Ttzm (40 μg/ml). GAPDH was used as a loading control. **b** Western blot analysis of protein extracts from NCI-N87 and MKN45 cells 48 h after transient transfected by MACC1 or vector, siMACC1 or siCtrl. GAPDH was used as a loading control. **c** Western blot analysis of protein extracts from NCI-N87 and MKN45 MACC1 overexpression or vector transfected cells, 24 h after treated with 1 μM MK2206. GAPDH was used as a loading control. **d** Glucose uptake (*red*) and lactate production (*blue*) of the cells was measured 24 h after NCI-N87 and MKN45 MACC1 overexpression plus or not plus treated with 1 μM MK2206. Cells were seeded in 96-well plates at 5 × 10^3^ cells/well, respectively. NC, NCI-N87, or MKN45 parental cells. Data represent mean ± SD of triplicate experiments, *P < 0.05, ^#^ *P* < 0.01, ^+^ *P* < 0.001.**e** MTT assays were performed 72 and 96 h after Ttzm (10 μg/ml) treatment of the indicated cells. Data represent mean ± SD of triplicate experiments relative to untreated cells. Inhibition of viability was compared between MACC1 and MACC1 + MK2206 group by multiple *t* test. *P < 0.05, ^#^ *P* < 0.01, ^+^ *P* < 0.001. **f** Western blot analysis of protein extracts from NCI-N87 and MKN45 cells 48 h after co-transfected with siMACC1 and Myr-AKT or siCtrl and Myr-AKT. GAPDH was used as a loading control. **g** Glucose uptake (*red*) and lactate production (*blue*) of the cells were measured 24 h after NCI-N87 and MKN45 siMACC1/siCtrl plus or not plus Myr-AKT co-transfected. Cells seeded in 96-well plates at 5 × 10^3^ cells/well, respectively. NC, NCI-N87, or MKN45 parental cells. Data represent mean ± SD of triplicate experiments, *P < 0.05, ^#^ *P* < 0.01, ^+^ *P* < 0.001. **h** MTT assays were performed 72 and 96 h after Ttzm (10 μg/ml) treatment of the indicated cells. Data represent mean ± SD of triplicate experiments relative to untreated cells. Inhibition of viability was compared between siMACC1 and siMACC1 + Myr-Akt group by Student’s *t* test.*P < 0.05, ^#^ *P* < 0.01, ^+^ *P* < 0.001

Trastuzumab could inhibit metabolism-regulating molecules such as PI3K and mTOR. Here, we found that it can also inhibit the AKT phosphorylation in NCI-N87 and MKN45 cell lines (Fig. 4a). Together with the results from prior studies (Figs. 1e and 2c), the AKT signaling pathway was activated and the Warburg effect was enhanced upon MACC1 upregulation in GC cells, meanwhile trastuzumab resistance was enhanced. Whereas, resistance to trastuzumab was attenuated in MACC1-downregulated groups, simultaneously, the phosphorylation of AKT decreased and the Warburg effect was suppressed (Figs. 1e and 2d). Thus, there are multirelationships between MACC1, the PI3K/AKT signaling pathway, the Warburg effect, and trastuzumab resistance in HER2-positive GC cells. Based on what we found, it suggests that there is a regulatory axis associated with trastuzumab resistance in HER2-positive GC cells: MACC1 enhanced the Warburg effect via the PI3K/AKT signaling pathway. To investigate this axis, we treated MACC1-upregulated NCI-N87 and MKN45 cell lines with MK2206, an AKT1/2/3 inhibitor. The results showed that MK2206 suppressed the expression of HK2 and LDHA (Fig. 4c) and decreased glucose uptake and lactate production (Fig. 4d), which were increased due to MACC1 upregulation. Moreover, after inhibiting the activity of PI3K/AKT signaling pathway by MK2206, the enhanced drug resistance was reversed (Fig. 4e).

To further confirm the regulatory pathway “MACC1-PI3K/AKT-Warburg-trastuzumab resistance,” the AKT constitutively activator Myr-AKT [27, 28] and MACC1 siRNA were applied to co-transfect NCI-N87 and MKN45 cell lines. The inhibited PI3K/AKT pathway was rescued as phosphorylation of AKT was enhanced (Fig. 4f). The Warburg effect was suppressed when MACC1 expression was disrupted by siRNA, but this effect was reversed when Myr-AKT was added, as evidenced by the increased expression of HK2 and LDHA (Fig. 4f). The decreased glucose uptake and lactate production caused by MACC1 downregulation were also reversed when the PI3K/AKT signaling pathway was constitutively activated (Fig. 4g). The enhanced cell inhibitory effect in response to trastuzumab caused by the downregulation of MACC1 was diminished when the PI3K/AKT signaling pathway was activated again (Fig. 4h). These consistent data demonstrate that inhibition of PI3K/AKT reverses MACC1-induced Warburg effect enhancement and trastuzumab resistance in GC cells. Accordingly, MACC1 enhanced trastuzumab resistance via regulation of the Warburg effect, in which the PI3K/AKT signaling pathway was mainly responsible for it.

### MACC1 induced trastuzumab resistance and enhanced the Warburg effect in vivo

**Fig. 5:**
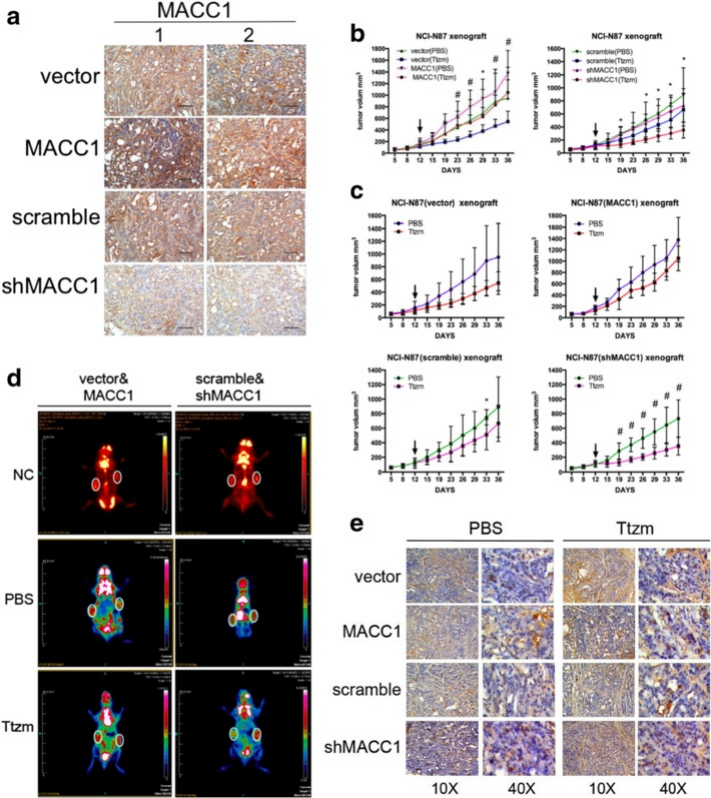
MACC1 induced trastuzumab resistance and enhanced the Warburg effect in vivo. **a** Representative images of MACC1 protein immunostaining in two xenografts tumor tissue. Anti-MACC1 immunohistochemistry of two NCI-N87 xenografts tumor tissue. *Black bar*, 50 μm. **b** Pre-established NCI-N87 MACC1 overexpression or vector tumor xenografts (*left*) and NCI-N87 MACC1 downregulation or scramble tumor xenografts (*right*) were treated with Ttzm (10 mg/kg, i.p., 2 times/wk × 3 weeks). *Arrow* means begin to treat drugs. Data are presented as the tumor volume (mm^3^), *n* = 6. Statistical analysis using the Student’s *t* test. *P < 0.05, ^#^ *P* < 0.01, ^+^ *P* < 0.001. **c** Pre-established NCI-N87 MACC1 overexpression or vector tumor xenografts (*upper*), and NCI-N87 MACC1 downregulation or scramble tumor xenografts (*below*) were treated with Ttzm (10 mg/kg, i.p., 2 times/wk × 3 weeks) or PBS (10 mg/kg, i.p., 2 times/wk × 3 weeks). *Arrow* means begin to treat drugs. Data are presented as the tumor volume (mm^3^), *n* = 6. Statistical analysis using the multiple *t* test. *P < 0.05, ^#^ *P* < 0.01, ^+^ *P* < 0.001. **d** MicroPET demonstrates that MACC1 overexpression promotes ^18^F-FDG uptake in mice bearing NCI-N87 xenograft. *R*, right (MACC1 overexpression or downregulation); *L*, left (vector or scramble). *White circles* present the tumor location. **e** Representative IHC TUNEL staining of tumor tissue from mice bearing NCI-N87 xenografts pretreatment with and Ttzm

To confirm the trastuzumab resistance promoter role of MACC1 in vivo, we established a BALB/c nude mouse xenograft model using NCI-N87 MACC1-overexpressing/silenced and their control cells. The tumor volume was measured twice a week until they reached the average volume (120 mm^3^). Subsequently, MACC1 expression was confirmed in xenografts using IHC staining (Fig. 5a).

Our previous results showed that MACC1 enhanced trastuzumab resistance in vitro. Here, to testify these results in vivo, we treated tumor xenografts with PBS as control or trastuzumab after the tumor had formed. Xenografts with MACC1 overexpression were more resistant to trastuzumab treatment than the vector group, whereas, were more sensitive in MACC1-downregulated xenografts than the scramble group (Fig. 5b). Compared to PBS, trastuzumab could inhibit the tumor growth more effectively when MACC1 was downregulated (Fig. 5c). Animal PET scanning demonstrated that ^18^F-FDG accumulation was markedly enhanced by MACC1 overexpression. Compared with PBS groups, ^18^F-FDG accumulation was inhibited in trastuzumab-treated groups more obviously in MACC1-silenced group rather than in scramble groups, but the effect of ^18^F-FDG accumulation mediated by trastuzumab did not show up when MACC1 overexpressing (Fig. 5d, Additional file 1: Figure S4). Taken together, these results suggested that MACC1 could induce trastuzumab resistance and enhance the Warburg effect in vivo.

We also investigated the apoptosis of tumor cells in xenografts, which showed that trastuzumab induced cell apoptosis when MACC1 was downregulated (Fig. 5e).

### Combination of trastuzumab and oxamate effectively inhibited cell growth and the Warburg effect in MACC1-overexpressing xenografts

**Fig. 6:**
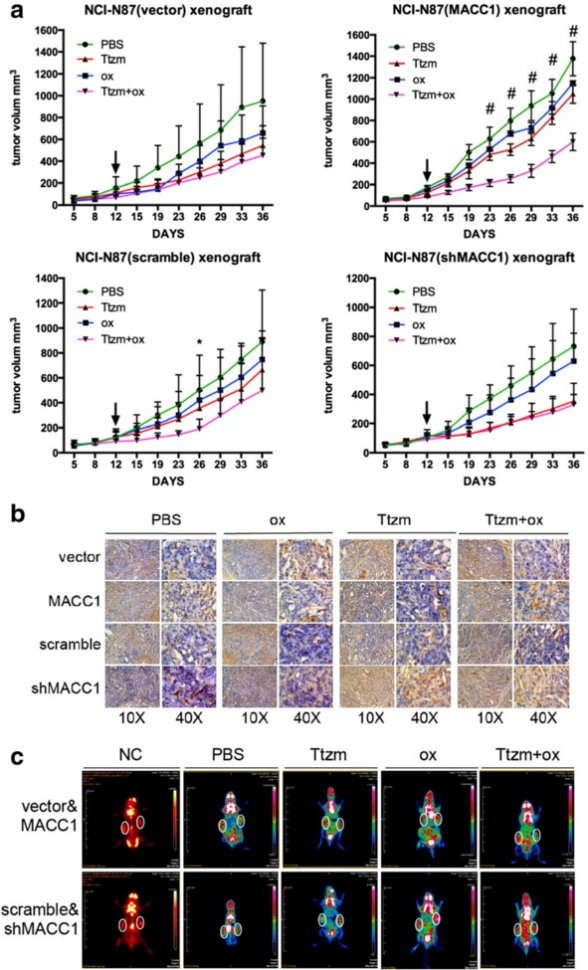
Combination of trastuzumab and oxamate effectively inhibited tumor growth and the Warburg effect in vivo. **a** Pre-established NCI-N87 tumor xenografts were treated with Ttzm (10 mg/kg,i.p., 2 times/wk × 3 weeks), OX (750 mg/kg,i.p.,daily for 21 days), or a combination of the agents. Arrow means begin to treat drugs. Data are presented as the tumor volume (mm^3^), *n* = 6. Statistical analysis of the differences of average tumor sizes in the Ttzm treatment group and the combination group was performed using the multiple *t* test. *P < 0.05, ^#^ *P* < 0.01, ^+^ *P* < 0.001. **b** Representative IHC TUNEL staining of tissue from mice bearing NCI-N87 xenografts pretreatment with PBS, OX, Ttzm, and Ttzm plus OX. **c** MicroPET demonstrates that trastuzumab plus oxemate inhibit ^18^F-FDG uptake most effectively in mice bearing NCI-N87 xenograft. *R*, right (MACC1 overexpression or downregulation); *L*, left (vector or scramble). *White circle* presents the tumor location

Based on our findings that trastuzumab combined with a glycolysis inhibitor synergistically inhibited the growth of both trastuzumab-sensitive and trastuzumab-resistant cells in vitro (Fig. 3d, e). To further confirm these combined effect in vivo, we treated tumor xenografts with trastuzumab (10 mg/kg, i.p., 2 times/wk × 3 weeks), oxamate (750 mg/kg, i.p., daily for 21 days), or a combination of the agents after tumor formation. The combination of trastuzumab with oxamate more efficiently inhibited the growth of tumors than the activity of any individual agent. The synergistic inhibitory effect was stronger in the MACC1-overexpressing group than the MACC1 downregulated and their control groups (Fig. 6a), which indicated that MACC1 might be a prognosis factor for the combined use of trastuzumab and glycolysis inhibitors in HER2-positive GC. Tumor cell apoptosis was also confirmed, which also followed the same trend (Fig. 6b).

Furthermore, animal PET scanning demonstrated that ^18^F^-^FDG accumulation was markedly inhibited by the combination of trastuzumab and oxamate compared to the outcomes of single drug groups in MACC1-overexpressing groups rather than the other ones (Fig. 6c, Additional file 1: Figure S5). The data of xenografts suggesting that the combination of trastuzumab with oxamate effectively inhibited tumor growth and the Warburg effect in vivo and MACC1 may demonstrate the synergistic inhibitory effect in HER2-positive GC. These results further support the finding that co-targeting of HER2 and the Warburg effect in MACC1-overexpressing HER2-positive GC cells contributed to overcoming trastuzumab resistance.

## Discussion

Trastuzumab in combination with chemotherapy has become the first-line treatment for advanced GC with HER2 overexpression [4, 29]. Although the response rates to this combination are much higher than those of chemotherapy alone, the effects are usually transitory, suggesting a high incidence of acquired resistance [4–6]. The supposed mechanisms of trastuzumab resistance presented so far include abrogation of productive drug-target contact through overexpression of MUC4 glycoprotein [30]; upregulation of target-like tyrosine kinase receptors or their ligands [31, 32]; alterations of target-downstream components in the PI3K/AKT signaling pathway such as PI3KCA [33] and PTEN [34]; cell reprogramming by deregulation of Bcl-2 [35], cycE [36], Mcl-1, and survivin [37]; and EMT transition [38]. Owing to the heterogeneous nature of tumors, different resistance mechanisms may coexist in the same patient; thus, targeting single mechanism is usually ineffective. To develop more powerful regimens to overcome drug resistance, signaling nodes which involved in multiple resistance mechanisms need to be identified.

As a pivotal oncogene for tumor progression influencing the HGF/c-MET pathway, MACC1, has been shown to participate in many biological mechanisms that produce poor clinical outcomes [39]. However, few studies have so far been explored the upstream mechanisms of MACC1, despite miR-338-3p, ORAI calcium release-activated calcium modulator 1 (Orai1), and stromal interacting molecule 1 (STIM1) being revealed as able to modulate MACC1 in GC [23, 40]. Previously, we found that MACC1 contributed to the poor prognosis of GC [21] and increased the resistance to metabolic stress by promoting the Warburg effect that consequently facilitated tumor progression [22], which elucidated its key role in signaling networks associated with GC. Besides, the Warburg effect was closely correlated to trastuzumab resistance and facilitated tumor progression [13, 26]. In preliminary experiment, we unexpectedly found that the expression of MACC1 protein was significantly increased in trastuzumab-resistant cells. On the basis of these findings, we hypothesized that MACC1 participated in trastuzumab resistance through regulating the Warburg effect. As the gene encoding the hepatocyte growth factor (HGF) receptor, MET, is a transcriptional target of MACC1 [20], the downstream signaling pathway of HGF-c-MET maybe involved in the mechanism of the“MACC1 regulating the resistant to trastuzumab” in gastric cancer cells. Since now, the PI3K/AKT [41, 42], RAS-RAF-MAPK [43, 44], and STAT3 [45] signaling pathway were identified as a regulatory axis in the resistance to trastuzumab in HER2-positive cancers. Our previous researches found that the PI3K/AKT pathway was involved in the resistant mechanisms in HER2-positive GC cells [18]. Together with MACC1-regulating cancer growth via the activation of the HGF/c-MET/PI3K/AKT signaling pathway [23, 46, 47], we purposed that MACC1 might induce trastuzumab resistance by regulating the Warburg effect via the PI3K/AKT signaling pathway. This research aimed to investigate the relationship between MACC1, the Warburg effect, and the PI3K/AKT signaling pathway in trastuzumab resistance in HER2-positive GC cells. These three targets may provide new routes for circumventing trastuzumab resistance and improving the therapeutic effects.

From our results, MACC1, PI3K/AKT signaling pathway, and the Warburg effect exerted critical function in trastuzumab-resistant GC cells. Here exits an effective pathway in trastuzumab resistance of HER2-positive GC cells: MACC1-PI3K/AKT-Warburg effect. Besides the cell viability and glycometabolism, we also detected cell apoptosis induced by trastuzumab in cells including MACC1-overexpressed plus or not plus PI3K-inhibited (by LY294002) cells, MACC1-silenced plus or not plus AKT-activated (by Myr-AKT) cells. When MACC1 was upregulated, the cell apoptosis caused by trastuzumab was inhibited, while the effect was reversed when LY294002 was added. When MACC1 was downregulated, the cell apoptosis caused by trastuzumab was enhanced, meanwhile, the effect was reversed when Myr-AKT was added (Additional file 1: Figure S6). The coupling of the MACC1, PI3K/AKT signaling, and Warburg effect is an important event that promotes survival of the resistant GC cells in the presence of trastuzumab.

Activation of the PI3K/AKT signaling pathway is known to mediate resistance to both molecularly targeted therapy and chemotherapy in various cancers [48]. Targeting MACC1 could induce inactivate the downstream signaling pathway such as PI3K/AKT. Interesting, we found that blockade of AKT activation inhibited the expression of MACC1 conversely; what is more, activated AKT also upregulated MACC1 expression (Fig. 4c, f). MACC1 has been reported as the upstream regulator of c-MET/AKT in hepatocellular cancer [46], cervical cancer [49], ovarian cancer [50], and gastric cancer [23]. On the contrary, whether MACC1 could be regulated by AKT-positive feedback loop has not been reported. Based on many AKT and its regulators and positive feedback phenomenon in tumor cells [51–53], it demonstrates a probable positive regulatory feedback loop between MACC1 and the PI3K/AKT signaling pathway, which needs to be testified and investigated deeply.

Warburg, in 1956, observed that many cancer cells used glycolysis more than mitochondrial oxidative phosphorylation for their energy requirements; this phenomenon is called “the Warburg Effect”[7]. The enzymes directly regulating glycolysis have also been implicated in promoting a drug-resistant phenotype. Targeting metabolic key enzymes can enhance therapeutic efficacy or combat drug resistance by promoting drug-induced apoptosis of cancer cells [54, 55]. Based on our results, the Warburg effect may contribute to chemoresistance or therapy failure in patients with GC, making the disease difficult to cure using a single agent. Targeting the Warburg effect improves the response to cancer therapeutics, and combining targeted drugs with cellular metabolism inhibitors may represent a promising strategy to overcome drug resistance in cancer therapy [25, 56]. A lot of tumor suppressors and oncoproteins, including the PI3K/AKT/mTOR signaling pathway, Myc, p53, and hypoxia-inducible factor-1 (HIF-1), have been reported to be involved in the regulation of the Warburg effect that favors tumor cell growth, proliferation, and stress resistance [57]. In this study, we demonstrated that trastuzumab and glycolysis inhibitors synergistically suppressed the growth and glycometabolism of HER2-positive GC cells in vitro and in vivo*.* More importantly, this combined use effectively inhibits the growth and Warburg effect of trastuzumab-resistant cancer cells, suggesting a potential benefit of this regimen in reversing trastuzumab resistance in HER2-positive GC.

LDHA is not necessary for normal tissue survival, as witnessed by the observation that humans with a hereditary deficiency of this LDH isoform do not show any symptom under normal circumstances [58]. Therefore, we chose oxamate, which specifically hinders enzymatic activity by competing with pyruvate, combined with trastuzumab to treat the xenografts. For the first time, we identified that MACC1 as the predictor for synergistically inhibitory effect of the combination of trastuzumab with oxamate in GC cells’ growth and glycolysis. These novel findings indicated that MACC1 promotes the Warburg effect via activation of the PI3K/AKT signaling pathway and contributes to the resistance of GC cells to trastuzumab. These in vitro and in vivo results indicated that MACC1 might be a selective factor in the use of co-targeting HER2 and glycolysis therapy in trastuzumab-resistant HER2-positive GC. We have provided new clues as to the mechanism by which the effect of the Warburg effect on trastuzumab-resistant GC cells is mediated. We also highlighted the involvement of MACCI in the regulation of the Warburg effect.

## Conclusions

**Fig. 7:**
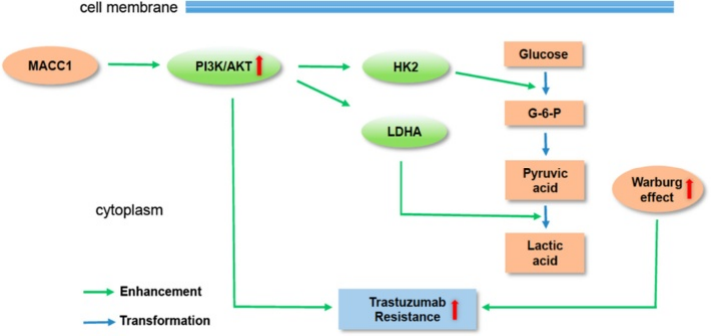
Diagram summarizing the proposed mechanism by which MACC1 drives resistance to trastuzumab. MACC1 promotes the Warburg effect through the PI3K/AKT signaling pathway. Both the activation of PI3K/AKT pathway and the enhancement of the Warburg effect induce the resistance to trastuzumab in HER2-positive GC

In this study, we demonstrated that MACC1 participated in trastuzumab resistance by promoting the Warburg effect, which was mainly through PI3K/AKT signaling pathway (Fig. 7). Importantly, we provide preclinical evidence indicating that co-targeting HER2 and glycolysis drastically benefits therapeutic outcomes for current anticancer therapy, suggesting that this combination may reverse trastuzumab resistance in patients with MACC1 overexpression and HER2-positive GC.

## Additional files

Additional file 1: Figures S1 to S6. Figure S1: The expression of proteins in GC cells and the sequences of ectopic MACC1 and shRNA. Figure S2: The combination of trastuzumab and glycolysis inhibitors synergisticly inhibit glycolysis in HER2 positive GC cells. Figure S3: The combination of trastuzumab and glycolysis inhibitors synergisticly inhibit glycolysis in HER2 positive GC cells. Figure S4: MACC1 enhanced the Warburg effect in vivo. Figure S5: Combination of trastuzumab and oxamate effectively inhibited the Warburg effect in vivo. Figure S6: The apoptosis of indicated cells after treated with Ttzm. (ZIP 38363 kb) Additional file 2: Tables S1 to S4. Table S1: IC50 of different GC cell lines. Table S2: IC50 and RI (resistance index) of MKN45 cells after treatment with trastuzumab at different inducing concentrations. Table S3: Fa and *CI* for trastuzumab and glucolysis inhibitor combinations on inhibition of cell viability. Table S4: Fa and *CI* for trastuzumab and glucolysis inhibitor combinations on inhibition of glucose uptake. (DOCX 45 kb)

## Abbreviations

MACC1: Metastasis associated with the colon cancer 1
GC: Gastric cancer
PI3K/AKT: Phosphatidylinositol 3-kinase/Protein kinase B
LDHA: Lactate dehydrogenase A
HK2: Hexokinase 2
Ttzm: Trastuzumab
OX: Oxamate
2-DG: 2-Deoxy-d-glucose
IC50: Half maximal inhibitory concentration
shRNA: Short hairpin RNA
siRNA: Small interfering RNA
Fa: Fractions affected
CI: Combination indices
