# Supplementary material for: A new mechanism of trastuzumab resistance in gastric cancer: MACC1 promotes the Warburg effect via activation of the PI3K/AKT signaling pathway
Source: J Hematol Oncol. 2016 Aug 31;9(1):76. doi: 10.1186/s13045-016-0302-1 (PMC5007850; doi:10.1186/s13045-016-0302-1)
Supplement: Additional file 1: — Figures S1 to S6. Figure S1: The expression of proteins in GC cells and the sequences of ectopic MACC1 and shRNA. Figure S2: The combination of trastuzumab and glycolysis inhibitors synergisticly inhibit glycolysis in HER2 positive GC cells. Figure S3: The combination of trastuzumab and glycolysis inhibitors synergisticly inhibit glycolysis in HER2 positive GC cells. Figure S4: MACC1 enhanced the Warburg effect in vivo. Figure S5: Combination of trastuzumab and oxamate effectively inhibited the Warburg effect in vivo. Figure S6: The apoptosis of indicated cells after treated with Ttzm. (ZIP 38363 kb) [file 13045_2016_302_MOESM1_ESM.zip › Legends of Supplementary Figures.docx]

**Supplementry Figure S1: The expression of proteins in GC cells and the sequences of ectopic MACC1 and shRNA. a.**Western blot analysis of MACC1 and HER2 expression in BGC823,MKN28,SGC7901,NCI-N87 and MKN45 cells.

GAPDH was used as a loading control**. b.** The ectopic MACC1 coding sequence and sequences of short hairpin RNA targeting MACC1**.**

**Supplementry Figure S2: The combination of trastuzumab and glycolysis**

**inhibitors synergisticly inhibit glycolysis in HER2 positive GC cells. a,b.** NCI-N87 and MKN45 parental(A) and trastuzumab resistance(B) cells were seeded in 96-well plates at 5×10^3^ cells/well.After 24 hours, cells were treated with the indicated concentration of Ttzm,2DG,OX,or Ttzm plus 2DG /OX and incubated for 24 hours, glucose uptake was determined. Data are presented as the percentage of glucose uptake inhibition measured in cells not treated with Ttzm nor 2-DG/OX.Data represent mean ± SD of triplicate experiments, **P*<0.05, ^#^*P*<0.01,^+^*P*<0.001.S,synergy (*CI*< 1.0).

**Supplementry Figure S3: The combination of trastuzumab and glycolysis**

**inhibitors synergisticly inhibit glycolysis in HER2 positive GC cells.a,b.**

Combination Index (*CI*) for experimental values of cell viability inhibiton by Ttzm

plus 2-DG/OX in NCI-N87, MKN45 parental (A) and trastuzumab resistant (B)cells,

as measured by Chou and Talalay method.

**Supplementry Figure S4: MACC1 enhanced the Warburg effect in vivo.**

MicroPET of mice bearing NCI-N87 xenograft after tumor reached the average

volume(120mm^3^). Vector (left), MACC1(right),scramble (left), shMACC1(right).

**Supplementry Figure S5:** **Combination of trastuzumab and oxamate effectively inhibited the Warburg effect in vivo**. MicroPET of mice bearing NCI-N87 xenograft after treated with indicated drugs. vector(left), MACC1(right),scramble (left),shMACC1(right).

**Supplementry Figure S6: The apoptosis of indicated cells after treated with Ttzm. a.** NCI-N87 cells were transfected with MACC1 over-expression /vector, MACC1 down-regulation/scramble, or were co-transfected with shMACC1 +Myr-

AKT/scramble +Myr-AKT,48 hours after transfection, cells were treated or not

treated with 1μM LY294002 for 24 hours, then, reated with Ttzm(10μg/ml) and

incubated for 48 hours, Flow cytometry of annexin V and PI-labeled all groups of

NCI-N87 cells.**b**.Curve graph of a.
