# Supplementary figures and images for: A new mechanism of trastuzumab resistance in gastric cancer: MACC1 promotes the Warburg effect via activation of the PI3K/AKT signaling pathway
Source: J Hematol Oncol. 2016 Aug 31;9(1):76. doi: 10.1186/s13045-016-0302-1 (PMC5007850; doi:10.1186/s13045-016-0302-1)

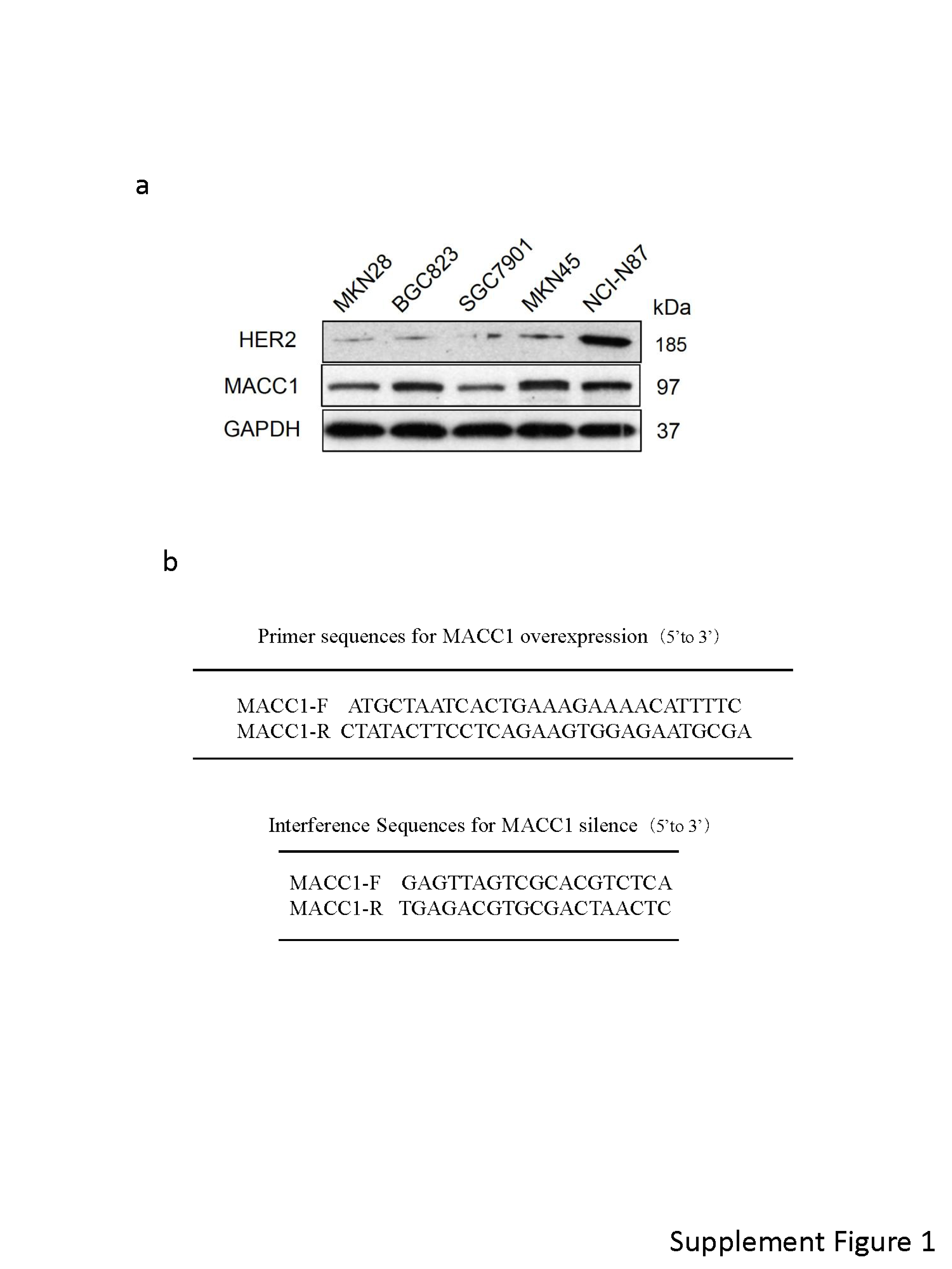

Supplement: Additional file 1: — Figures S1 to S6. Figure S1: The expression of proteins in GC cells and the sequences of ectopic MACC1 and shRNA. Figure S2: The combination of trastuzumab and glycolysis inhibitors synergisticly inhibit glycolysis in HER2 positive GC cells. Figure S3: The combination of trastuzumab and glycolysis inhibitors synergisticly inhibit glycolysis in HER2 positive GC cells. Figure S4: MACC1 enhanced the Warburg effect in vivo. Figure S5: Combination of trastuzumab and oxamate effectively inhibited the Warburg effect in vivo. Figure S6: The apoptosis of indicated cells after treated with Ttzm. (ZIP 38363 kb) [file 13045_2016_302_MOESM1_ESM.zip › Additional file1 Supplementry Figure S1.tiff]

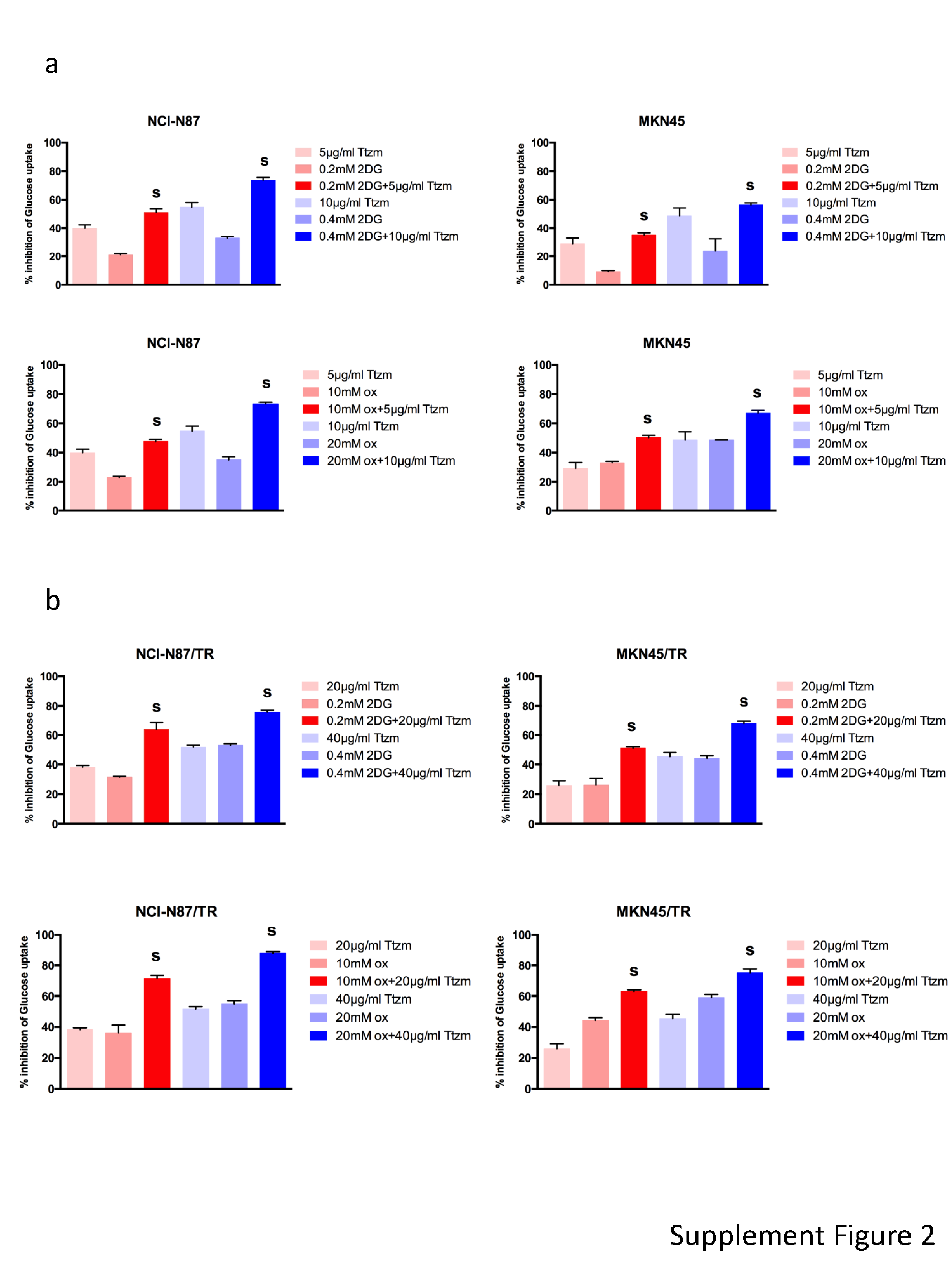

Supplement: Additional file 1: — Figures S1 to S6. Figure S1: The expression of proteins in GC cells and the sequences of ectopic MACC1 and shRNA. Figure S2: The combination of trastuzumab and glycolysis inhibitors synergisticly inhibit glycolysis in HER2 positive GC cells. Figure S3: The combination of trastuzumab and glycolysis inhibitors synergisticly inhibit glycolysis in HER2 positive GC cells. Figure S4: MACC1 enhanced the Warburg effect in vivo. Figure S5: Combination of trastuzumab and oxamate effectively inhibited the Warburg effect in vivo. Figure S6: The apoptosis of indicated cells after treated with Ttzm. (ZIP 38363 kb) [file 13045_2016_302_MOESM1_ESM.zip › Additional file1 Supplementry Figure S2.tiff]

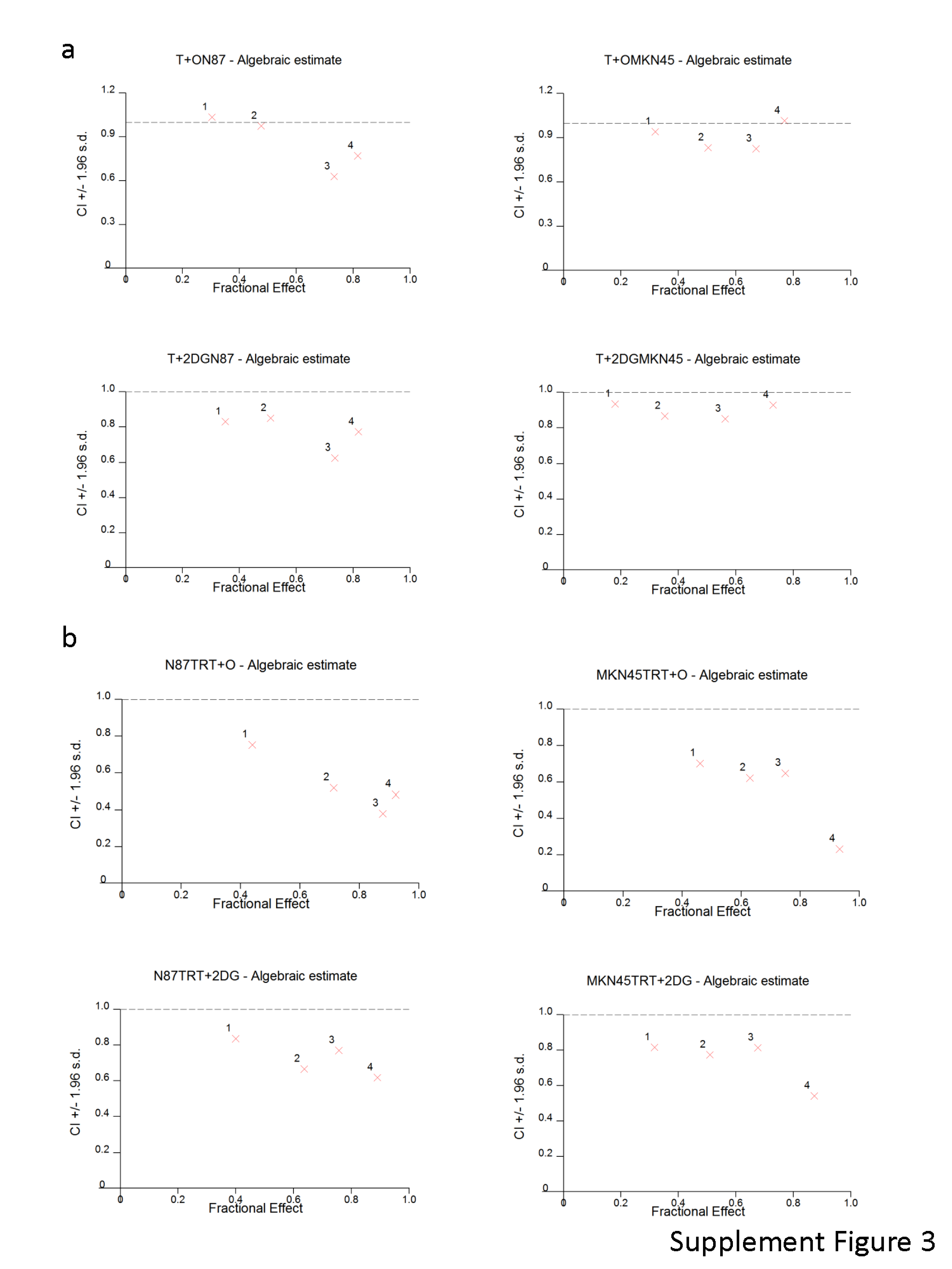

Supplement: Additional file 1: — Figures S1 to S6. Figure S1: The expression of proteins in GC cells and the sequences of ectopic MACC1 and shRNA. Figure S2: The combination of trastuzumab and glycolysis inhibitors synergisticly inhibit glycolysis in HER2 positive GC cells. Figure S3: The combination of trastuzumab and glycolysis inhibitors synergisticly inhibit glycolysis in HER2 positive GC cells. Figure S4: MACC1 enhanced the Warburg effect in vivo. Figure S5: Combination of trastuzumab and oxamate effectively inhibited the Warburg effect in vivo. Figure S6: The apoptosis of indicated cells after treated with Ttzm. (ZIP 38363 kb) [file 13045_2016_302_MOESM1_ESM.zip › Additional file1 Supplementry Figure S3.tiff]

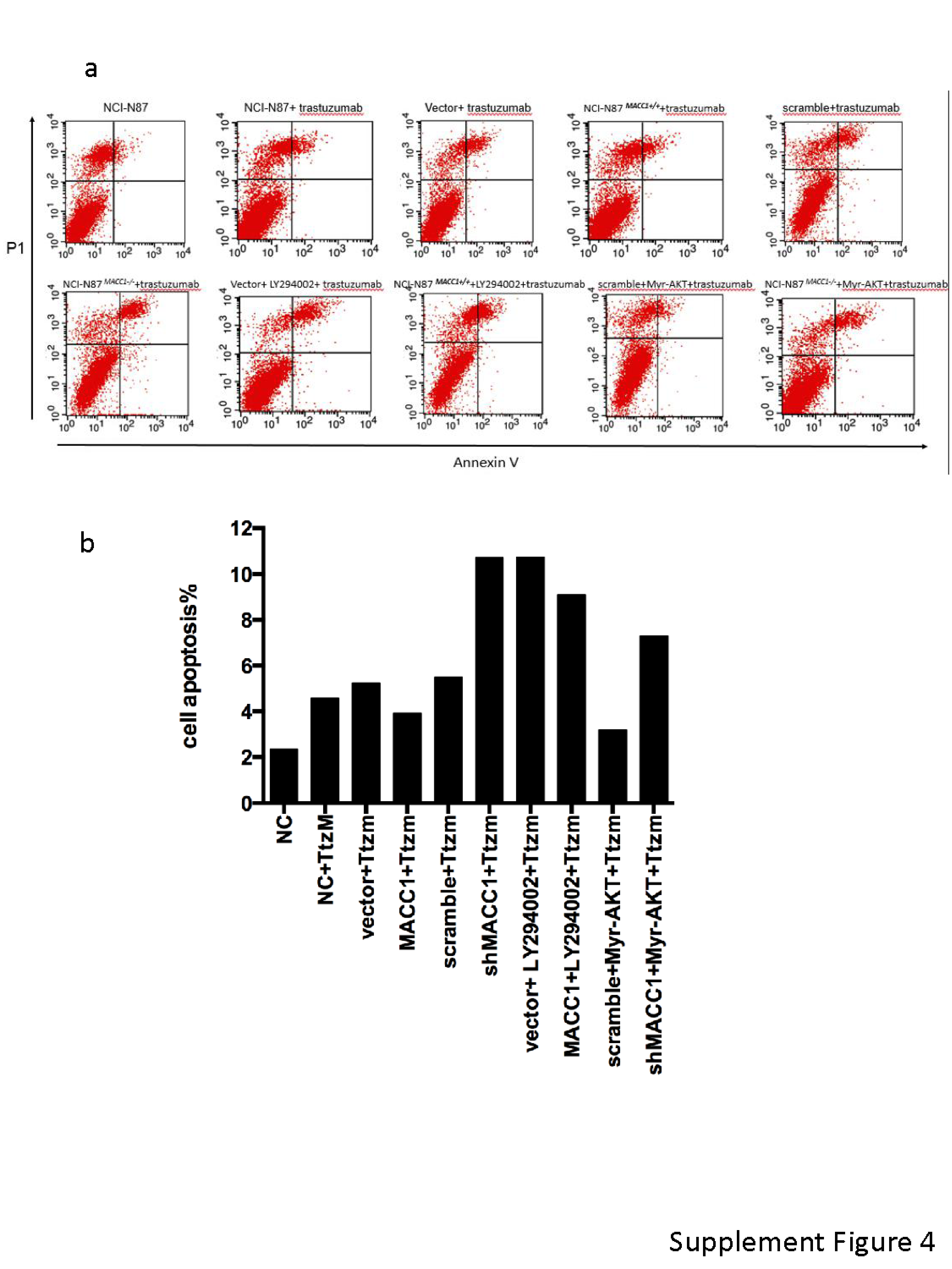

Supplement: Additional file 1: — Figures S1 to S6. Figure S1: The expression of proteins in GC cells and the sequences of ectopic MACC1 and shRNA. Figure S2: The combination of trastuzumab and glycolysis inhibitors synergisticly inhibit glycolysis in HER2 positive GC cells. Figure S3: The combination of trastuzumab and glycolysis inhibitors synergisticly inhibit glycolysis in HER2 positive GC cells. Figure S4: MACC1 enhanced the Warburg effect in vivo. Figure S5: Combination of trastuzumab and oxamate effectively inhibited the Warburg effect in vivo. Figure S6: The apoptosis of indicated cells after treated with Ttzm. (ZIP 38363 kb) [file 13045_2016_302_MOESM1_ESM.zip › Additional file1 Supplementry Figure S6.tiff]
