# Supplementary material for: A new mechanism of trastuzumab resistance in gastric cancer: MACC1 promotes the Warburg effect via activation of the PI3K/AKT signaling pathway
Source: J Hematol Oncol. 2016 Aug 31;9(1):76. doi: 10.1186/s13045-016-0302-1 (PMC5007850; doi:10.1186/s13045-016-0302-1)
Supplement: Additional file 2: — Tables S1 to S4. Table S1: IC50 of different GC cell lines. Table S2: IC50 and RI (resistance index) of MKN45 cells after treatment with trastuzumab at different inducing concentrations. Table S3: Fa and CI for trastuzumab and glucolysis inhibitor combinations on inhibition of cell viability. Table S4: Fa and CI for trastuzumab and glucolysis inhibitor combinations on inhibition of glucose uptake. (DOCX 45 kb) [file 13045_2016_302_MOESM2_ESM.docx]

**Supplementary Table S1: IC50 of different GC cell lines.**

| **Cell lines** | **Trastuzumab(μg/ml)** | **oxemate(mM)** | **2DG(mM)** |
| --- | --- | --- | --- |
| NCI-N87 | 24.407 | 71.438 | 1.821 |
| MKN45 | 30.297 | 38.835 | 0.260 |
| NCI-N87(MACC1) | 50.369 |  |  |
| NCI-N87(vector) | 22.051 |  |  |
| NCI-N87(shMACC1) | 12.975 |  |  |
| NCI-N87(scramble) | 21.077 |  |  |
| MKN45(MACC1) | 199.136 |  |  |
| MKN45(vector) | 41.068 |  |  |
| MKN45(shMACC1) | 12.402 |  |  |
| MKN45(scramble) | 49.422 |  |  |
| NCI-N87/TR | 243.978 | 42.228 | 0.894 |
| MKN45/TR | 296.258 | 20.002 | 0.120 |

**Supplementary Table S2: IC50 and RI(Resistance Index)of MKN45 cells after treatment with trastuzumab at different inducing concentrations.**

| **Trastuzumab(μg/ml)** | **IC50(μg/ml)** | **RI** |
| --- | --- | --- |
| 0 | 30.297 |  |
| 10 | 33.068 | 1.091 |
| 20 | 40.213 | 1.327 |
| 40 | 45.466 | 1.501 |
| 80 | 48.513 | 1.601 |
| 160 | 51.004 | 1.683 |
| 320 | 55.814 | 1.842 |
| 640 | 68.781 | 2.270 |
| 1280 | 134.019 | 4.424 |
| 1600 | 198.028 | 6.536 |
| 2000 | 236.700 | 7.813 |
| 2500 | 296.258 | 9.778 |

**Supplementary Table S3: Fa and *CI* for trastuzumab and glucolysis inhibitor combinations on inhibition of cell viability.**

| **Range of CI** | **Symbol** | **Description** |
| --- | --- | --- |
| <0.1 | ＋＋＋＋＋ | Very strong synergism |
| 0.1-0.3 | ＋＋＋＋ | Strong synergism |
| 0.3-0.7 | ＋＋＋ | Synergism |
| 0.7-0.85 | ＋＋ | Moderate synergism |
| 0.85-0.90 | ＋ | Slight synergism |
| 0.90-1.10 | ± | Nearly additive |
| 1.10-1.20 | － | Slight antagonism |
| 1.20-1.45 | －－ | Moderate antagonism |
| 1.45-3.3 | －－－ | Antagonism |
| 3,3-10 | －－－－ | Strong antagonism |
| >10 | －－－－－ | Very strong antagonism |

Supplementary Table S3-1:Symbol and description of Combination index(*CI*).

| **Cell lines** | **Trastuzumab**  **(μg/ml)** | **Oxemate**  **(mM)** | **Fa** | ***CI*** |
| --- | --- | --- | --- | --- |
| NCI/N87 | \| 2.5 \| \| --- \| \| 5 \| \| 10 \| \| 20 \| \| 40 \| | \| 6 \| \| --- \| \| 12.5 \| \| 25 \| \| 50 \| \| 100 \| | 0.195605  0.312071  0.511762  0.635893  0.8034 | 0.895  0.926  0.763  0.984  0.775 |
| MKN45 | \| 2.5 \| \| --- \| \| 5 \| \| 10 \| \| 20 \| \| 40 \| | \| 6 \| \| --- \| \| 12.5 \| \| 25 \| \| 50 \| \| 100 \| | 0.34391  0.449011  0.578911  0.694223  0.779133 | 0.524  0.514  0.439  0.395  0.397 |
| NCIN87/TR | \| 40 \| \| --- \| \| 80 \| \| 160 \| \| 320 \| \| 640 \| | \| 6 \| \| --- \| \| 12.5 \| \| 25 \| \| 50 \| \| 100 \| | 0.44398  0.592877  0.66349  0.81749  0.900283 | 0.417  0.253  0.303  0.168  0.117 |
| MKN45/TR | \| 5 \| \| --- \| \| 10 \| \| 20 \| \| 40 \| \| 80 \| | \| 6 \| \| --- \| \| 12.5 \| \| 25 \| \| 50 \| \| 100 \| | 0.384017  0.613197  0.723053  0.874097  0.931307 | 0.484  0.380  0.461  0.356  0.379 |

Supplementary Table S3-2: Complete values for Fractions affected (Fa) and Combination index (CI) for trastuzumab and oxamate combinations(inhibitions of cell viability).Calculations by CalcuSyn software using the method of Chou and Taladay.

| **Cell lines** | **Trastuzumab**  **(μg/ml)** | **2DG**  **(mM)** | **Fa** | ***CI*** |
| --- | --- | --- | --- | --- |
| NCI/N87 | \| 2.5 \| \| --- \| \| 5 \| \| 10 \| \| 20 \| \| 40 \| | \| 0.1 \| \| --- \| \| 0.2 \| \| 0.4 \| \| 0.8 \| \| 1.6 \| | 0.198939  0.312071  0.511762  0.635893  0.8034 | 0.793  0.870  0.763  0.930  0.824 |
| MKN45 | \| 2.5 \| \| --- \| \| 5 \| \| 10 \| \| 20 \| \| 40 \| | \| 0.1 \| \| --- \| \| 0.2 \| \| 0.4 \| \| 0.8 \| \| 1.6 \| | 0.394853  0.546283  0.723593  0.899474  0.93725 | 0.736  0.754  0.655  0.358  0.421 |
| NCIN87/TR | \| 40 \| \| --- \| \| 80 \| \| 160 \| \| 320 \| \| 640 \| | \| 0.1 \| \| --- \| \| 0.2 \| \| 0.4 \| \| 0.8 \| \| 1.6 \| | 0.35705  0.465883  0.657472  0.77065  0.893183 | 0.763  0.839  0.605  0.596  0.389 |
| MKN45/TR | \| 5 \| \| --- \| \| 10 \| \| 20 \| \| 40 \| \| 80 \| | \| 0.1 \| \| --- \| \| 0.2 \| \| 0.4 \| \| 0.8 \| \| 1.6 \| | 0.26964  0.413853  0.55027  0.67962  0.767555 | 0.240  0.162  0.129  0.104  0.101 |

Supplementary Table S3-3:Complete values for Fractions affected (Fa) and Combination index (CI) for trastuzumab and 2DG combinations(inhibitions of cell viability).Calculations by CalcuSyn software using the method of Chou and Taladay.

**Supplementary Table S4: Fa and *CI* for trastuzumab and glucolysis inhibitor combinations on inhibition of glucose uptake.**

| **Cell lines** | **Trastuzumab**  **(μg/ml)** | **Oxemate**  **(mM)** | **Fa** | ***CI*** |
| --- | --- | --- | --- | --- |
| NCI/N87 | 2.5  5  10  20 | 5  10  20  40 | 0.303333  0.476667  0.733333  0.816667 | 1.036  0.974  0.630  0.770 |
| MKN45 | 2.5  5  10  20 | 5  10  20  40 | 0.32  0.503333  0.67  0.77 | 0.941  0.833  0.825  1.016 |
| NCIN87/TR | 10  20  40  80 | 5  10  20  40 | 0.44  0.713333  0.88  0.923333 | 0.752  0.517  0.381  0.481 |
| MKN45/TR | 10  20  40  80 | 5  10  20  40 | 0.46  0.63  0.75  0.933333 | 0.701  0.623  0.649  0.232 |

Supplementary Table S4-1: Complete values for Fractions affected (Fa) and Combination indices (CI) for trastuzumab and oxamate combinations(inhibitions of Glucose uptake by cells). Calculations by CalcuSyn software using the method of Chou and Taladay.

| **Cell lines** | **Trastuzumab**  **(μg/ml)** | **2DG**  **(mM)** | **Fa** | ***CI*** |
| --- | --- | --- | --- | --- |
| NCI/N87 | 2.5  5  10  20 | 0.1  0.2  0.4  0.8 | 0.35  0.51  0.736667  0.818333 | 0.830  0.851  0.626  0.773 |
| MKN45 | 2.5  5  10  20 | 0.1  0.2  0.4  0.8 | 0.18  0.353333  0.563333  0.73 | 0.934  0.867  0.851  0.928 |
| NCIN87/TR | 10  20  40  80 | 0.1  0.2  0.4  0.8 | 0.4  0.636667  0.756667  0.89 | 0.836  0.666  0.77  0.619 |
| MKN45/TR | 10  20  40  80 | 0.1  0.2  0.4  0.8 | 0.316667  0.551  0.676667  0.873333 | 0.817  0.775  0.814  0.542 |

Supplementary Table S4-2: Complete values for Fractions affected (Fa) and Combination indices (CI) for trastuzumab and 2DG combinations(inhibitions of Glucose uptake by cells). Calculations by CalcuSyn software using the method of Chou and Taladay.
